# Successful Treatment of Annular Atrophic Lichen Planus With Adalimumab

**DOI:** 10.1002/ccr3.70036

**Published:** 2024-12-31

**Authors:** Joe Khodeir, Paul Ohanian, Marielena Ohanian

**Affiliations:** ^1^ Department of Dermatology at Saint Georges Hospital University Medical Center University of Balamand, Faculty of Medicine and Medical Sciences Balamand Lebanon; ^2^ Department of Family Medicine at Saint Georges Hospital University Medical Center University of Balamand, Faculty of Medicine and Medical Sciences Balamand Lebanon; ^3^ Department of Geriatric Medicine, Nimes Hospital University Medical Center University of Balamand, Faculty of Medicine and Medical Sciences Balamand Lebanon

**Keywords:** adalimumab, annular atrophic lichen planus, ischemic stroke, recalcitrant lichen planus

## Abstract

Adalimumab may be an effective treatment for resistant annular atrophic lichen planus, especially in patients with prior treatment failures and contraindications to other systemic therapies. This case underscores the need for exploring systemic options in challenging cases of this rare lichen planus variant.

## 1 Introduction

Annular atrophic lichen planus (AALP) is an exceedingly rare variant of lichen planus, characterized by annular plaques with central atrophy and a reduction of elastin fibers in the superficial dermis. AALP typically presents as chronic, pruritic, violaceous lesions that can be refractory to conventional treatments. The etiology and pathogenesis of AALP remain poorly understood, largely due to the limited number of cases reported in the literature. While lichen planus is often associated with mucosal involvement, the occurrence of mucosal lesions in AALP is uncommon, and systemic associations are rarely documented. The management of AALP is challenging, as it frequently does not respond to standard therapies such as topical corticosteroids or immunosuppressants. Adalimumab, a monoclonal antibody that inhibits the tumor necrosis factor‐alpha (TNF‐α), is commonly used in dermatology to treat chronic inflammatory skin conditions such as psoriasis and hidradenitis suppurativa to reduce inflammation and skin lesions [1].

In this case report, we present a 56‐year‐old female with AALP and a history of ischemic cerebrovascular accident (CVA), whose condition improved significantly with adalimumab treatment after failing multiple other therapies. This case highlights the challenges in managing AALP and the potential benefits of systemic therapy.

## 2 Case History/Examination

**FIGURE 1:**
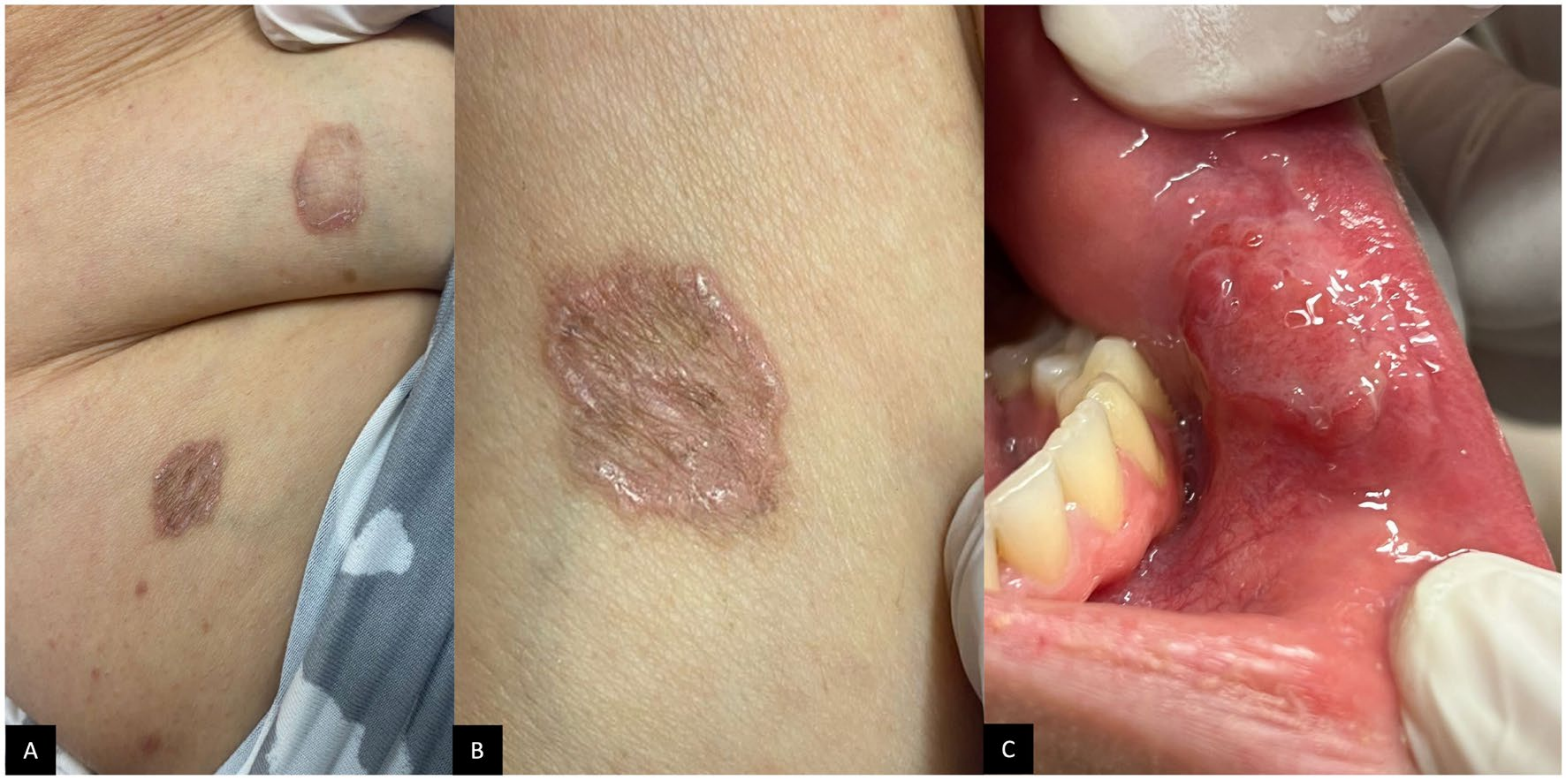
(A) Anular atrophic lichen planus lesions on the abdomen and thigh. (B) Well‐demarcated annular violaceous lesion with raised borders and central atrophy. (C) Oral mucosal ulcer on lower lip mucosa.

This is the case of a 56‐year‐old female with a history of ischemic cerebrovascular accident (CVA) 3 years ago and a 5‐year history of annular atrophic lichen planus (AALP), who presented to our dermatology clinic with non‐resolving pruritic cutaneous lesions. Despite consultation with multiple dermatologists and undergoing various treatments, including topical steroids, topical tacrolimus, oral hydroxychloroquine, oral steroids, and oral acitretin, the patient experienced minimal improvement. The diagnosis of AALP was initially confirmed by a biopsy and re‐confirmed upon presentation at our clinic. Physical examination revealed annular violaceous lesions with raised borders and central atrophy (Figure 1B) distributed over the abdomen, back, arms, and thighs bilaterally (Figure 1A). Additionally, a painful, non‐healing oral mucosal ulcer on the lower lip, persisting for 6 months, was observed (Figure 1C). No other mucosal, genital lesions, or nail involvement were present.

## 3 Methods (Differential Diagnosis, Investigations, and Treatment)

**FIGURE 2:**
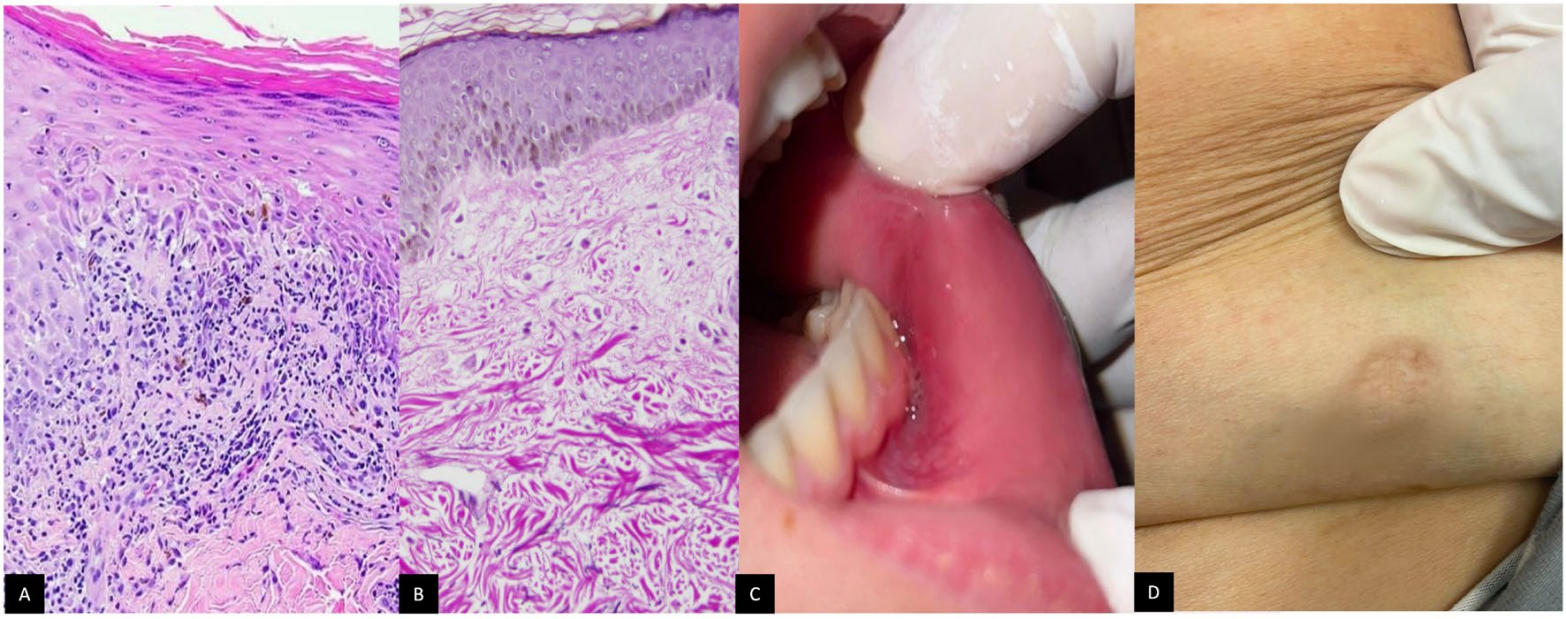
(A) Band‐like lymphocytic infiltrate at dermo‐epidermal junction, hypergranulosis, and necrotic keratinocytes (HE stain ×200). (B) The loss of elastic fibers in upper dermis (on Verhoeff‐van Gieson stain ×200). (C) The complete resolution of oral ulcer. (D) Cutaneous lesion remission with mild residual hyperpigmentation and atrophy.

Biopsies taken from both cutaneous and mucosal lesions confirmed AALP showing band‐like lymphocytic infiltrate at dermo‐epidermal junction, hypergranulosis, and necrotic keratinocytes with loss of elastic fibers in upper dermis on Verhoeff‐van Gieson stain (Figure 2A,B). In addition, hepatitis C virus serology was negative. Due to the patient's history of ischemic stroke, the use of oral JAK inhibitors or cyclosporine was avoided. Laboratory investigations, including complete blood count, C‐reactive protein, serum protein electrophoresis, complete liver profile, viral serologies, chest x‐ray, and cardiac ultrasound were all normal. Consequently, we initiated treatment with adalimumab, administering an 80 mg loading dose followed by 40 mg biweekly.

## 4 Results (Outcome and Follow‐Up)

Remarkably, after 8 weeks of therapy, both the cutaneous lesions (Figure 2D) and the mucosal ulcer (Figure 2C) showed significant improvement. At the 4‐month follow‐up, the patient achieved complete remission, her quality of life improved substantially as the pruritus subsided and the painful oral ulcer healed. During that period, follow‐up laboratory investigations were all normal. This treatment regimen was maintained, as no side effects were observed over 6 months.

## 5 Discussion

AALP is the rarest variant of lichen planus, distinguished by a reduction of elastin fibers in the superficial dermis. Due to the limited number of reported cases, the pathogenesis of AALP remains poorly understood. However, it is well recognized that this variant is notoriously difficult to treat, with topical steroids often proving ineffective [2]. Mucosal involvement is uncommon in this variant, with only one case reported by Zhang et al., who described AALP associated with mucosal and nail lesions [3].

AALP has also been reported in association with Sneddon's syndrome (a neurocutaneous vasculopathy characterized by the combination of livedo racemosa and recurrent cerebrovascular events), where a patient exhibited livedo reticularis, raynaud's phenomenon, and ischemic stroke. The authors suggested that an abnormal production of elastolytic enzymes might be a shared pathomechanism contributing to both dermal elastolysis and arteriolar damage [4].

Our patient experienced an ischemic CVA at the early age of 53, without any major risk factors such as hypertension, dyslipidemia, smoking, or family history of cardiovascular diseases. We propose that AALP may extend beyond the skin as a systemic inflammatory disorder with potential effects on other organs. The destruction of elastic fibers in the skin, characteristic of this variant [2], could similarly occur in the elastic fibers of cerebral vessels, potentially leading to ischemic CVA. Moreover, several studies have demonstrated an association between lichen planus and an increased risk of cardiovascular disease [5, 6, 7, 8, 9].

Given these considerations, we believe that the treatment of AALP should be systemic rather than solely local, reflecting the disease's systemic nature. In our patient with a history of ischemic stroke, oral JAK inhibitors and other immunosuppressives, such as cyclosporine, were not favored due to safety concerns. The production or release of TNF‐α within lichen planus lesions may significantly influence the immunopathogenesis of the disease [10]. Based on a few case reports showing successful treatment with adalimumab in recalcitrant mucosal, genital and cutaneous lichen planus cases [11], we initiated adalimumab therapy. Remarkably, our patient responded well to the treatment, making this the first reported case of adalimumab being used successfully in the management of annular atrophic lichen planus.

Other biologics used successfully in the treatment of mucocutaneous lichen planus included secukinumab, brodalumab and secukinumab in cases refractory to steroids, calcineurin inhibitors, hydroxychloroquine, acitretin, and methotrexate [12, 13].

## 6 Conclusion

This case highlights the difficulty of treating AALP with conventional therapies and demonstrates the potential effectiveness of systemic treatment with adalimumab. The significant improvement in our patient after failing multiple other treatments underscores the need for alternative approaches in managing this rare variant. Further research is necessary to explore the underlying mechanisms of AALP, particularly the loss of elastic fibers, and to develop optimal management strategies for this challenging variant of lichen planus.

## Author Contributions

**Joe Khodeir:** conceptualization, investigation, validation, writing – original draft, writing – review and editing. **Paul Ohanian:** conceptualization, methodology, writing – original draft. **Marielena Ohanian:** supervision, validation.

## Consent

Written informed consent was taken from the patient to publish their data and clinical figures.

## Conflicts of Interest

The authors declare no conflicts of interest.

## Data Availability

The data used to support the findings of this study are included within the article.
